# UK *BRCA* mutation testing in patients with ovarian cancer

**DOI:** 10.1038/bjc.2015.396

**Published:** 2015-12-15

**Authors:** Angela George

**Affiliations:** 1 The Royal Marsden NHS Foundation Trust, Fulham Road, London SW3 6JJ, UK

**Keywords:** *BRCA* mutation, ovarian cancer, guidelines, *BRCA1*, *BRCA2*, *BRCA* testing

## Abstract

Despite the increasing clinical importance of germline *BRCA* mutation status in managing women with ovarian cancer, few patients are currently being tested. The traditional means of selecting patients for *BRCA* mutation testing using restrictive criteria will miss many women with a mutation. To expand access to testing and streamline the testing process, several centres in the UK have been developing new models for *BRCA* testing. Trials with these integrated models involving closer collaborations between genetics and oncology services are now under way. In addition to testing for *BRCA* mutations, there is also increasing interest in testing for other genes associated with a predisposition to ovarian cancer. Advances in next-generation sequencing technology have resulted in the development of comprehensive genetic testing panels for use in the research and diagnostic settings. Interest is also increasing in expanding testing for somatic mutations in ovarian cancer, particularly for genes such as *BRCA1* and *BRCA2*, whereby mutations may allow more patients to benefit from targeted agents, including poly(ADP-ribose) polymerase inhibitors. In this review, the issues of who should be offered testing, how testing could be delivered, when testing should occur and the technology and costs associated with genetic testing are addressed.

The decisions about who should undertake testing for germline *BRCA1* and *BRCA2* mutations, as well as when and how it should be carried out, are becoming increasingly important for ovarian cancer patients in the UK. Over the last few years, greater public awareness of the genetic component of breast and ovarian cancer has resulted in increasing patient demand for testing. This, combined with an expanding range of clinical implications for ovarian cancer patients found to carry a *BRCA1* or *BRCA2* mutation, has presented a central role for genetic testing. Recent studies have suggested that approximately 15% of all ovarian cancer patients harbour a germline mutation in *BRCA1* and *BRCA2*, few of whom are currently being offered testing (Pal *et al*, 2005; Walsh *et al*, 2011; Zhang *et al*, 2011; Alsop *et al*, 2012).

Access to genetic testing for ovarian cancer patients has traditionally been limited to those who meet specific criteria, largely for the purpose of familial cancer risk assessment. This dates back to the resource-intensive nature of older genetic testing methods, which were very time consuming and expensive to undertake. Advances in sequencing technology have led to next-generation sequencing (NGS), which offers fast, efficient, high-throughput testing at a considerably lower cost than with older methods (Bentley *et al*, 2008; Harismendy *et al*, 2009). It also means that results can consistently be provided within a clinically useful timeframe, allowing their incorporation into treatment decisions, such as choice of chemotherapy or eligibility for trials. The availability of this technology also opens up opportunities to re-evaluate traditional means of selecting patients for testing, particularly how well these criteria perform in differentiating between patients who should and should not be offered testing.

In the UK, there are no standard guidelines for testing patients with ovarian cancer for germline mutations of *BRCA1*/*2*, with practice varying by region. Guidelines do exist for patients with breast cancer, whereby the National Institute for Health and Care Excellence (NICE) recommends that all women with a 10% chance of harbouring a *BRCA* mutation should be offered testing, and testing should be considered down to a threshold of 5% (NICE, 2013). Following the publication of these guidelines, many regions have considered offering testing to ovarian cancer patients meeting the 10% threshold. However, the difficulty lies in deciding how to determine that threshold. A number of risk prediction models have been developed to estimate the risk of an individual, all of which require family history details on which to base risk assessments. These models, such as BOADICEA or BRCAPRO, can be cumbersome to use, particularly in a busy clinic (Fischer *et al*, 2013). An alternative is a validated scoring system such as the Manchester Score. These can be rapidly assessed during clinic consultations to determine if an individual meets testing thresholds (Evans *et al*, 2004); however, they also require multiple *BRCA*-related cancers to be present within the family for the threshold to be met. Use of such models or scoring systems was recommended by NICE as an acceptable way of assessing an individual’s risk of harbouring a *BRCA* mutation (NICE, 2013).

The selection of patients for testing has long relied on the presence of a strong family history of breast and ovarian cancer. It is now clear from a number of studies of ovarian cancer patients, unselected for family history that this criterion will result in substantial numbers of those with a *BRCA* mutation being missed. Møller *et al* (2007) tested all women presenting to their unit with ovarian cancer and reported a *BRCA* mutation rate of 23%. Of these, only one-third qualified for testing based on their family history. Alsop *et al* (2012) found that 13% of women with non-mucinous ovarian cancer in their Australian study carried a *BRCA* mutation, with 44% of carriers reporting no family history of *BRCA*-associated cancers. Similar mutation rates have been reported in women without a family history in multiple European, Canadian and American studies. These findings have led to several centres in the USA, Canada and the UK offering *BRCA* testing to all women with non-mucinous ovarian cancers (Metcalfe *et al*, 2009). This approach will detect many more women with *BRCA* mutations who would not have been offered testing using a selective approach.

The optimal time to test for a *BRCA* mutation has never been agreed. Testing of women during first-line treatment allows incorporation of mutation status into future treatment decisions without having to wait for results, which could potentially delay treatment. This is particularly relevant for areas where the time from referral to availability of results remains in excess of 6 months. However, this approach would leave a large subset of patients untested: those on long-term follow-up and those with relapsing disease who have not previously been offered testing. Such patients would need to be identified and offered testing during their routine follow-up. An alternative would be to test women only when there is an immediate difference in treatment choices. However, this approach would mean that many patients would miss out on testing, either because their disease has not relapsed or because the length of time for testing in some areas would delay treatment well beyond a clinically desirable timeframe. Long-term survivors of ovarian cancer would still benefit from testing, especially with regard to being informed of their second cancer risk and ensuring that they receive appropriate screening and advice (Domchek *et al*, 2013).

As the need for identifying *BRCA* mutations in patients with ovarian cancer is increasing, several hospitals in England have been implementing genetic testing models to allow *BRCA* testing to become a part of the routine clinical care of patients with ovarian cancer (Table 1).

Cambridge University Hospitals initiated the Genetic Testing in Epithelial Ovarian Cancer (GTEOC) study in July 2013, to explore the feasibility and acceptability of offering direct testing for *BRCA* mutations to all women who are diagnosed with ovarian cancer. The study asked all patients who were diagnosed with serous or endometrial epithelial ovarian cancer within 12 months of the initiation of the study, irrespective of their age and family history of cancer, whether they would consent to *BRCA* testing. Initial findings from this study showed that the population-based genetic testing approach used appeared to be acceptable to patients, and was less resource intensive than standard practice, whereby all patients have a full assessment by the genetics department prior to testing (Tischkowitz *et al*, 2014).

The Royal Marsden Hospital has developed an ‘oncogenetic’ testing model, implemented in July 2013, to identify *BRCA* mutations in patients with ovarian cancer. Oncology clinicians have been trained and certified to allow them to obtain consent from patients for testing. Patients with non-mucinous ovarian cancer or who have both ovarian cancer and another primary tumour (any age) are offered *BRCA* testing at their appointment with the oncologist. The patient and the oncology clinician receive the results from the genetics department, and those patients who carry a *BRCA* mutation then attend a genetics appointment with their results to allow detailed discussion and to have *BRCA* testing offered for their relatives. In addition, patients can contact or be referred to the genetics department at any time at their own request or at the discretion of their oncologist. This oncogenetic model of identifying patients with a *BRCA* mutation has provided a flexible, patient-centred, impartial, high-throughput approach, which has resulted in considerable time and cost savings compared with a standard genetics referral (George *et al*, 2014).

In November 2013, Scotland updated the Scottish Intercollegiate Guidelines Network (SIGN) guidelines on the management of epithelial ovarian cancer (SIGN 135) to include *BRCA* testing as standard for all patients with ovarian cancer (Scottish Intercollegiate Guidelines Network (SIGN), 2013). The guideline states that: (1) all women with non-mucinous ovarian or fallopian tube cancer should be offered *BRCA1* and *BRCA2* mutation testing; (2) women with ovarian cancer who have a family history of breast, ovarian or colon cancer should have a genetic risk assessment; and (3) *BRCA1* and *BRCA2* mutation analysis should be considered in a family where there is a 10% or greater risk of a mutation being present. The SIGN guidelines state that close collaboration between primary care and specialist cancer genetics services should be encouraged, to enable efficient genetic cancer risk assessments in individuals who are at medium or high risk.

The information provided from the small number of English hospitals and the Scottish guidelines described here demonstrates that there is currently no consensus for genetic testing of patients with ovarian cancer to identify a *BRCA* mutation. Even in those with clear, open criteria, it is likely that patients who do meet the criteria are not referred for genetic testing. Such under-referral of patients has been frequently reported in studies at specialist centres such as the MD Anderson Cancer Center in Texas, USA. It has also been reported in areas where genetic testing is more widely available, such as British Columbia and Ontario in Canada, where women can be referred for testing based on histology alone. Despite this, reports indicate that only 20% of eligible women are referred for testing. The uptake rate among those who are referred for testing is high, indicating that it is not patient reluctance that limits testing. This suggests that it is not only the eligibility criteria, but the entire attitude towards testing for germline mutations that needs to be considered.

For a number of years, the ‘gold standard’ for *BRCA* mutation testing has involved a combination of Sanger sequencing and multiplex ligation-dependent probe amplification (MLPA). Direct Sanger sequencing allows identification of small variants (such as the deletion or insertion of single bases); whereas MLPA identifies large variants, such as the deletion or duplication of one or more exons. However, these methods are time consuming and costly, as they require the gene to be divided into small fragments, each of which is individually amplified and sequenced (Ruiz *et al*, 2014). More recently, high-throughput methods of NGS have been developed. These allow massive parallel sequencing of multiple genes; from multi-gene panels to whole genomes in a single run (Bentley, 2006). This technology has been rapidly adopted in research settings and is now used to perform diagnostic testing in several UK laboratories.

In addition to faster turnaround times for testing, another major potential advantage in the use of NGS is a reduction in the cost of performing *BRCA* testing. The standard cost of a *BRCA* test in the UK is currently £530 (NHS, 2014). There are several different NGS platforms available at present, all of which have slight variations in requirements and output. Each platform has variable costs per sample and costs for the sequencing equipment, but overall, the *BRCA* testing costs are lower using high-throughput NGS. This change results from higher levels of automation and far less laboratory technician time required to prepare and run samples. However, all platforms also require significant bioinformatic input for analysis and interpretation of the sequencing data. This has been a major limiting factor for laboratories considering moving to NGS, as few have such support available. Wider availability of cheaper testing may also increase demand for testing, increasing the need for integration between oncology and clinical genetics services.

Ovarian cancer is a genetically heterogeneous disease, with germline mutations in *BRCA1*, *BRCA2*, *MLH1*, *MSH2*, *MSH6*, *PMS2*, *RAD51D*, *RAD51C*, *BRIP1* and *PALB2* all associated with an inherited predisposition (Senter *et al*, 2008; Bonadona *et al*, 2011; Loveday *et al*, 2011; Pelttari *et al*, 2011; Rafnar *et al*, 2011; Loveday *et al*, 2012; Turnbull *et al*, 2014; Xiao *et al*, 2014). Mosaic mutations in the *PPM1D* gene have also been linked to ovarian cancer (Ruark *et al*, 2013). Of these, *BRCA1* and *BRCA2* appear to account for approximately two-thirds of germline mutations in ovarian cancer, with smaller contributions from the remaining genes (Walsh *et al*, 2011). This relative heterogeneity of ovarian cancer beyond *BRCA1* and *BRCA2* makes it ideally suited to either panel testing or exome testing, whereby comprehensive testing of multiple genes in parallel is performed. The sequential testing of multiple ovarian cancer genes is cumbersome and expensive and cannot be performed in a clinically relevant timeframe, leaving parallel sequencing the only viable alternative if all genes are to be evaluated in patients. This may soon become desirable, with genes such as *RAD51D* and *PALB2* shown to cause similar *in vivo* sensitivity to poly(ADP-ribose) polymerase (PARP) inhibitors as that demonstrated in *BRCA*-deficient cells (Loveday *et al*, 2011; Turnbull *et al*, 2014). Interest in these genes is likely to intensify if they are found to influence response to targeted treatments and chemotherapy.

A range of multi-gene panels has been developed for use in the diagnostic or research setting, although use of these panels is not currently available within the UK National Health Service. One of the major difficulties with cancer panels to date is the reporting of mutations in cancer patients for genes in which there is no evidence of a causal link, such as *MRE11A* in ovarian cancer (Pennington *et al*, 2014). As more people with malignancies are tested for a wide variety of genes, it is increasingly likely that pathogenic mutations will be reported in genes that have previously been assessed or reported for that tumour type. It is possible that in some cases this will be found to be causally linked to the cancer, but in many cases it is likely to reflect the population frequency of such mutations. Determining which of those found are causative and how such variants should be reported will be a major challenge of the cancer panel era.

Somatic mutations in a range of different genes have been associated with each subtype of epithelial ovarian cancer. There is already interest in therapeutic exploitation of such somatic mutations, with trials investigating the role of drugs targeting *BRAF*, *MTOR*, *MEK* and *AKT* in ovarian cancer. There is also interest in identifying patients with somatic mutations in DNA repair genes such as *BRCA1* and *BRCA2*, as they may demonstrate similar synthetic lethality to PARP inhibitors as that shown with germline mutations. Somatic mutations have been reported in DNA repair genes in approximately 9% of ovarian cancer, while *BRCA1* promoter hypermethylation is reported in approximately 10% of high-grade serous and high-grade endometrioid ovarian cancer (Esteller *et al*, 2000; Cancer Genome Atlas Research Network, 2011; Pennington *et al*, 2014).

For years, the primary advantage in ovarian cancer patients undergoing genetic testing was the identification of at-risk family members, who would then choose risk-reducing interventions to modify their future risk of cancer. There are now clear differences in the behaviour, response to treatment options and prognosis of ovarian cancer between those with and without a *BRCA1* or *BRCA2* mutation. The emphasis should therefore be placed back on the individual with ovarian cancer to undergo testing to inform their management. Here, the integrated oncogenetic pathways have an advantage, with the oncology teams able to discuss the extent of the impact that knowledge of mutation status will have on an individual’s care when obtaining informed consent. In order to move forward to patient-centred, personalised cancer treatment in the era of targeted agents, genetic testing must now be considered an important part of the diagnostic process. In summary, it would be appropriate for all non-mucinous ovarian cancer patients to be offered *BRCA* mutation testing at the time of their diagnosis, to inform and enable the most appropriate treatment decisions to be made for each individual patient.

## Figures and Tables

**Table 1:** Local hospital (England) and Scottish Health Board guidelines for *BRCA* testing procedures in patients with ovarian cancer (as of October 2014)

**Centre**	***BRCA*** **mutation testing process**
Cambridge University Hospitals	*BRCA* testing standard as part of the GTEOC study
The Royal Marsden Hospital	*BRCA* testing standard as part of the oncogenetic model, began in July 2013 Fully integrated service
University Hospitals Birmingham	*BRCA* testing of all newly diagnosed high-grade serous ovarian cancer patients began in April 2014 Fully integrated service
The Royal Liverpool University Hospitals	Testing of all high-grade serous ovarian cancer patients at diagnosis Testing offered to prevalent patients upon entering the clinic Training/consenting courses available for oncologists Fully integrated service
Oxford University Hospitals	*BRCA* testing on request for patients with high-grade serous ovarian cancer
Central Manchester University Hospitals	*BRCA* testing on request for selected patients Currently (October 2014) not a fully integrated service but aiming to achieve this status; currently consent can only be obtained through a specialist genetics clinic
Scotland	All ovarian cancer patients are tested for *BRCA* mutations at diagnosis (funded by the Scottish Health Board) Consent for *BRCA* mutation testing is obtained by oncologists Fully integrated service

Abbreviation: GTEOC, Genetic Testing in Epithelial Ovarian Cancer.
